# Efficacy and safety of nebulized drugs in the treatment of non-severe *mycoplasma* pneumoniae pneumonia in children - a network meta-analysis

**DOI:** 10.3389/fphar.2025.1587152

**Published:** 2025-09-02

**Authors:** Li Zeng, Hanlin Ye, Qiu Zhang, Ting Xu, Hong Xu, Dan Bi

**Affiliations:** ^1^ Department of Pharmacy, The Third People’s Hospital of Yibin, Yibin, China; ^2^ Department of Pharmacy, The Second People’s Hospital of Yibin, Yibin, China

**Keywords:** *mycoplasma* pneumoniae pneumonia(MPP), children, nebulized drugs, azithromycin (AZM), network meta-analysis

## Abstract

**Objective:**

To systematically evaluate the efficacy and safety of commonly used nebulized drugs as adjuvant treatment for non-severe *mycoplasma* pneumoniae pneumonia (MPP) in children, providing an evidence-based reference for clinical medication.

**Methods:**

A computerized search of major Chinese and English literature databases was conducted to collect randomized controlled trials (RCTs) that utilized nebulized drugs in conjunction with azithromycin (AZM) or AZM alone in children with MPP. Following literature screening, the quality of the included studies was evaluated using the risk of bias assessment tool as recommended by the Cochrane Handbook. Outcome data for each measurement were extracted, and a network meta-analysis was conducted using Stata 17.0.

**Results:**

A total of 79 RCTs involving 7,712 patients and 9 interventions were included. The network meta-analysis indicated that all nebulized drugs combined with AZM markedly improved clinical efficacy compared to AZM alone. Among these, the combination of nebulized budesonide plus terbutaline demonstrated superior efficacy and safety as adjunctive therapy for non-severe MPP. Furthermore, Ambroxol combined with AZM was particularly effective in shortening the duration of clinical symptoms such as fever and lung rales. The combinations of terbutaline or budesonide with AZM significantly improved pulmonary function.

**Conclusion:**

Nebulized drug adjuvants to AZM therapy enhance treatment efficacy in children with non-severe MPP without increasing the incidence of adverse events. However, there is a need for more extensive, higher-quality clinical studies to overcome the limitations due to the low quality of some included articles.

## 1 Introduction

*Mycoplasma* pneumoniae pneumonia (MPP) is the most common form of atypical pneumonia in children, accounting for approximately 37.5%–48.4% of pneumonia cases in hospitalized pediatric patients (Tang et al., 2023; Gao et al., 2019). Mild MPP is typically characterized by fever, cough, and inflammatory changes in the bronchi or alveoli. However, if left untreated, MPP can trigger excessive inflammation and immune responses, leading to compromised respiratory function and, in severe cases, multi-organ damage, which can be fatal. Current therapeutic protocols prioritize macrolide antibiotics, with azithromycin (AZM) serving as the cornerstone of first-line treatment for mild pediatric MPP (Rizzato et al., 1995). However, reliance on AZM alone may not adequately alleviate persistent symptoms such as cough and wheezing, potentially resulting in prolonged disease duration or treatment failure (Zhang et al., 2024). Therefore, there is an urgent need to explore additional therapeutic strategies to mitigate immune damage, alleviate symptoms, and improve outcomes for affected children.

Currently, nebulization therapy has been increasingly utilized for various respiratory diseases in children. In the case of MPP, the combination therapy of AZM and nebulized drugs provides synergistic effects via multiple mechanisms, including antibacterial action, anti-inflammatory responses, and enhanced ventilation. Firstly, AZM exerts systemic anti-infective effects by inhibiting bacterial ribosomal protein synthesis (Heidary et al., 2022), while nebulized drugs act directly on the local respiratory tract, rapidly alleviating symptoms through their anti-inflammatory and spasmolytic properties (Martin and Finlay, 2015). The complementary targets of these two therapies simultaneously address symptom control and the underlying causes of infection. Secondly, nebulized drugs enhance mucociliary clearance, which improves antibiotic penetration efficiency at lesion sites and further amplifying antibacterial efficacy. Finally, AZM diffuses into infected tissues via the bloodstream, whereas nebulized drugs deliver a high local concentration to the airway mucosa with minimal systemic absorption. The metabolic pathways of these treatments avoid mutual interference, reducing the risks of systemic side effects. Studies have demonstrated that nebulized drugs can improve respiratory tract inflammation, alleviate cough symptoms, shorten the duration of illness, and enhance clinical prognosis in children, providing robust support for the clinical management of MPP infections (Yan et al., 2025; Bian et al., 2017). Furthermore, their non-invasive nature and suitability for long-term use make them particularly appropriate for pediatric patients, greatly enhancing treatment acceptability (Hickey and Mansour, 2019). These theoretical advantages provide a solid scientific foundation and broad clinical prospects for nebulized drug therapy in children with MPP.

In recent years, several studies have demonstrated the potential of various monotherapy and combination nebulized drug treatment regimens (Yan et al., 2025; Direkwattanachai et al., 2023). However, there is a notable lack of comprehensive research that explores the efficacy and safety comparisons among different nebulization strategies. This study employs a network meta-analysis to comprehensively evaluate the efficacy and safety of various nebulized drug treatment regimens combined with AZM for the treatment of non-severe MPP in children, aiming to assist clinicians in determining a more suitable nebulization treatment plan.

## 2 Methods

### 2.1 Search strategy and selection criteria

This network meta-analysis was conducted in accordance with the Preferred Reporting Items for Systematic Reviews and Meta-Analyses (PRISMA) Statement (Hutton et al., 2015) and was registered with the International Prospective Register of Systematic Reviews (PROSPERO NO: CRD42025638663).

We selected relevant studies published from the establishment of the databases to 30 December 2024, by CNKI, VIP, Wan Fang, CBM, PubMed, Cochrane Library, Embase and Web of Science. The search terms included: *mycoplasma* pneumonia, atomization, nebulization therapy, children. (details of the search strategies are provided in Additional File 1).

### 2.2 Inclusion and exclusion criteria

The inclusion criteria adhered to the PICOS format, which encompasses participants, interventions, comparisons/controls, outcomes, and study design. These criteria were as follows: 1) Participants Children (≤14 years of age) were diagnosed with MPP based on laboratory and imaging examinations, without any underlying diseases, such as bronchial asthma, or other infectious lesions. The diagnosis of MPP was established through clinical symptoms, chest imaging, and microbiological testing, which included culture, polymerase chain reaction testing, or serologic testing (Respiratory et al., 2023). 2) Intervention AZM combined with nebulized drug therapy (AZM was administered intravenously, orally, or sequentially, and nebulized drug therapy included the use of single or multiple drugs). 3) Control The control group received either AZM sequential therapy alone or in combination with nebulized drugs. 4) Outcomes The primary outcomes assessed were clinical efficacy rate and the incidence of adverse events. Secondary outcomes included the disappearance time of fever, the disappearance time of cough, the disappearance time of lung rales, and pulmonary function. The definition of clinical efficacy is detailed in Additional File 2, referencing relevant consensus or research (Kuzman et al., 2005; Weiss et al., 2019; Association, 2004). Pulmonary function parameters included forced vital capacity (FVC), the ratio of forced expiratory volume in one second/forced vital capacity (FEV1/FVC) and peak expiratory flow (PEF). 5) Study design Only RCTs published in Chinese and English were included.

### 2.3 Exclusion criteria

The exclusion criteria were as follows: studies that failed to meet the predefined inclusion criteria were excluded, particularly those focusing on refractory or severe MPP infections. Characteristic manifestations of severe MPP in children included persistent high fever (≥39 °C) lasting for five or more days, presentation of respiratory distress, chest pain or hemoptysis, presence of severe allergic diseases, immunocompromised status or chronic airway/pulmonary diseases, as well as general data indicating severe disease (Respiratory et al., 2023). Additionally, studies on drug treatments outside of the primary interventions were excluded, such as antipyretic and antitussive medications, glucocorticoids, or traditional Chinese medicine. Studies lacking complete texts or those with incomplete data or duplicate reports were also not considered.

### 2.4 Data extraction and quality evaluation

Two investigators (ZL and XH) independently screened the literature based on established inclusion and exclusion criteria, followed by a cross-check of their results. In instances of disagreement during the screening process, a third party was consulted to assist in the judgment and resolve the issue through discussion. Endnote literature management software and excel were utilized to manage and extract study data, including the number of included cases, intervention measures, course of treatment, treatment cycles, and outcome indicators. All included articles underwent a comprehensive evaluation using the Cochrane Risk of Bias Assessment Tool (Cumpston et al., 2019), which assessed six aspects: randomization method, allocation concealment, blinding, completeness of outcome data, selective reporting, and other sources of bias. The Grading of Recommendations Assessment, Development and Evaluation (GRADE) framework was employed to rank the certainty of evidence for each pair of comparisons, categorizing them into four levels: high, medium, low, or very low.

### 2.5 Statistical analysis

Statistical analysis was performed using Stata 17 software. The relative odds ratio (OR) and 95% credible intervals served as effect indicators for binary outcomes. For continuous variables, the mean difference (MD) and 95% credible intervals were employed. A probability value of *p* < 0.05 was defined as statistically significant. Stata 17 software was also used to plot the evidence network, and consistency tests were conducted when closed loops were present in the evidence network (Higgins et al., 2012). The surface under the cumulative ranking curve (SUCRA) for each intervention was used to reflect the efficacy of different treatment regimens; a SUCRA value closer to 100% indicated a higher likelihood that the treatment regimen demonstrated optimal efficacy. Additionally, funnel plots of clinical efficacy rates and the incidence of adverse events were constructed to assess potential publication bias or the influence of small-sample sizes.

## 3 Results

### 3.1 Search results

**FIGURE 1:**
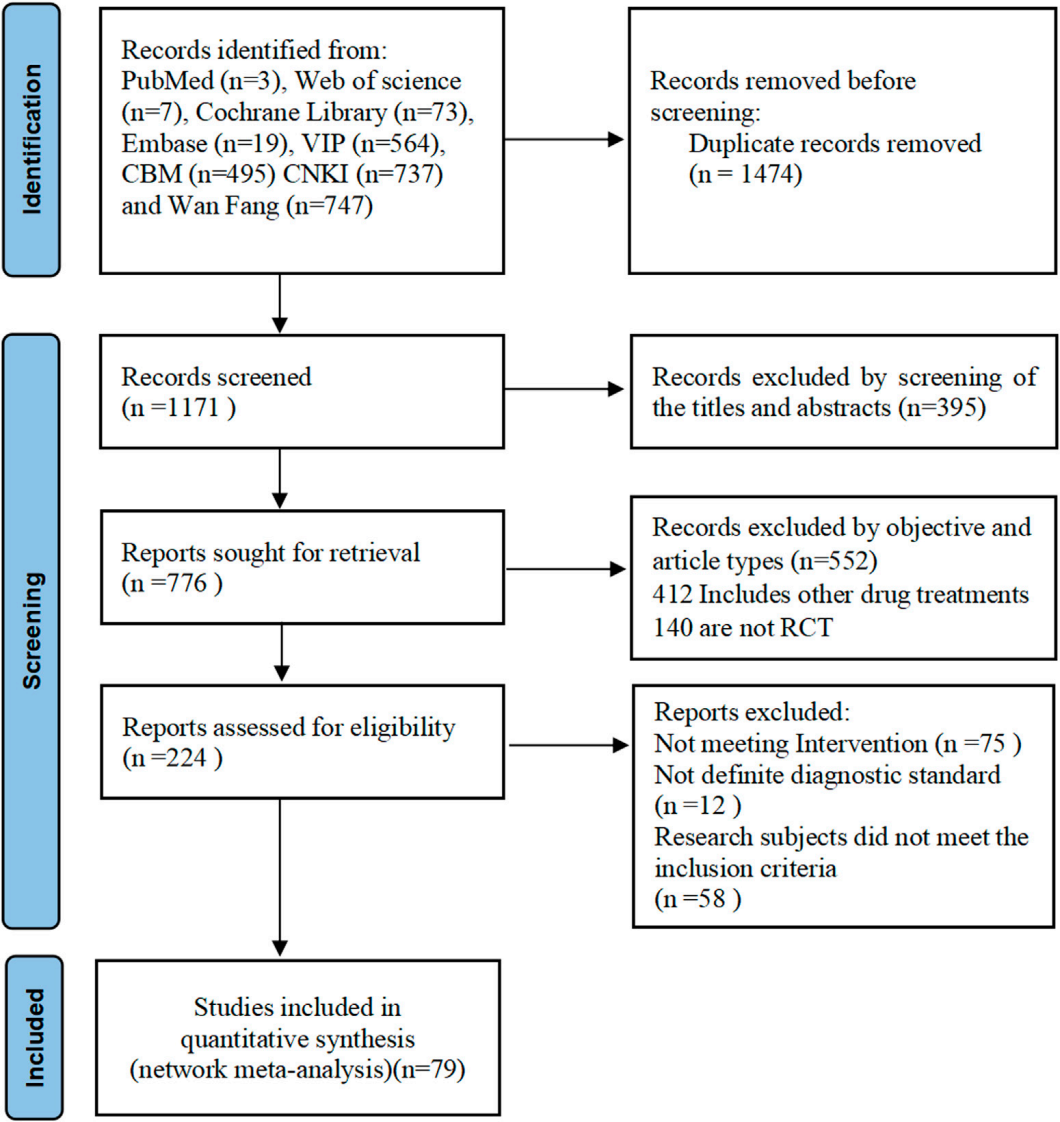
Flow chart of literature screening.

A total of 2,645 relevant articles were initially selected based on the search terms. After removing 1,474 duplicate articles using EndNote software, the titles and abstracts of the remaining 1,171 studies were reviewed, resulting in the exclusion of 395 irrelevant studies. Subsequently, 776 articles were reviewed in a second phase, and their full texts were evaluated according to the established inclusion and exclusion criteria. Ultimately, 79 clinical studies were included in the current systematic review. The screening process is illustrated in Figure 1.

### 3.2 Study characteristics

This study included a total of 79 RCTs involving 7,712 patients, which assessed 9 interventions: AZM, AZM combined with nebulized drug (including budesonide, acetylcysteine, terbutaline, ambroxol, salbutamol, budesonide + terbutaline, budesonide + salbutamol and ipratropium + salbutamol). The basic characteristics of the included studies are summarized in Table 1. All RCTs were Chinese literature, and the data between groups were comparable.

**TABLE 1:** General characteristics of the included RCTs.

Study	Cases		Sex (M/F)		Average age		Intervention measures		Treatment course(d)	Outcome
	E	C	E	C	E	C	E	C		
Wu and Zhang, (2024)	47	47	21/26	25/22	5.7 ± 1.2	5.4 ± 1.6	AZM + Budesonide (1 mg,bid)	AZM	14	①②③④⑤⑥
Gao, (2024)	50	50	25/25	24/26	6.1 ± 0.5	5.9 ± 0.3	AZM + Budesonide (0.5 mg,bid)	AZM	7	①②③④⑥
Chen, (2022a)	40	40	17/23	19/21	4.67 ± 1.45	4.12 ± 1.34	AZM + Budesonide (0.5 mg,bid)	AZM	7	①②⑥
Liu, (2021)	48	48	28/20	26/22	—	—	AZM + Budesonide (0.5 mg,bid)	AZM	7	①②⑥
Feng, (2023)	39	39	22/17	20/19	5.12 ± 0.22	6.32 ± 0.35	AZM + Budesonide (1 mg,bid)	AZM	7	①③④⑥
Zhang, (2023a)	40	40	24/16	25/15	8.45 ± 1.14	7.46 ± 2.23	AZM + Budesonide (1 mg,bid)	AZM	14	①⑤
Yin, (2016)	35	33	20/15	19/14	6.5 ± 3.9	5.5 ± 6.1	AZM + Budesonide (0.5 mg,bid)	AZM	14	①
Yan, (2024)	52	52	27/25	28/24	5.31 ± 1.76	5.08 ± 1.82	AZM + Budesonide (0.5 mg,bid)	AZM	14	①②③④⑤⑥
Ma, (2024)	30	30	14/16	13/17	4.63 ± 0.24	4.66 ± 0.21	AZM + Budesonide (1 mg,bid)	AZM	7	②③④⑤
Liu, (2022)	118	117	59/58	62/56	7.09 ± 1.08	7.03 ± 0.22	AZM + Budesonide (1 mg,bid)	AZM	14	②③④⑤⑥
Lin, (2021)	82	81	40/42	41/40	8.22 ± 3.82	8.33 ± 3.34	AZM + Budesonide (1 mg,bid)	AZM	7	①②③④⑤
Lin et al. (2019)	41	41	21/20	22/19	3.6 ± 0.4	3.5 ± 0.4	AZM + Budesonide (1 mg,bid)	AZM	12	①②④
Li, (2021)	140	140	69/71	76/64	6.4 ± 0.9	6.3 ± 1.2	AZM + Budesonide (0.5 mg,bid)	AZM	7	①②③④⑥
Xue, (2024)	43	43	23/20	22/21	6.25 ± 2.08	6.21 ± 2.06	AZM + Budesonide (0.5 mg,bid)	AZM	10	①②③④⑤
Chen and Li, (2023)	29	29	14/15	16/13	4.83 ± 3.94	5.54 ± 3.56	AZM + Budesonide (2 mg,bid)	AZM	10	①②③④
Liu, (2015)	38	38	22/16	24/14	6.0 ± 3.9	5.7 ± 3.7	AZM + Budesonide (0.5 mg,bid)	AZM	21	①②③④⑥
Cai et al. (2016)	30	30	15/15	17/13	6.61 ± 2.06	7.69 ± 2.66	AZM + Budesonide (0.5 mg,bid)	AZM	14	②③④
Zhou et al. (2022)	51	50	30/21	30/20	7.79 ± 2.12	7.84 ± 2.05	AZM + Budesonide (1 mg,bid)	AZM	14	①②③④
Yin, (2019)	40	32	22/18	18/14	6.79 ± 0.84	6.81 ± 0.68	AZM + Budesonide (1 mg,bid)	AZM	14	③④⑥
Ye et al. (2020)	50	50	31/19	29/21	4.62 ± 0.43	4.83 ± 0.62	AZM + Budesonide (bid)	AZM	7	①②③④⑥
Xu, (2019)	49	49	29/20	28/21	5.63 ± 0.79	5.69 ± 0.81	AZM + Budesonide (0.5 mg,bid)	AZM	7	①②③④⑥
Wu and Yang (2024)	50	50	33/17	21/19	4.63 ± 1.44	4.48 ± 1.32	AZM + Budesonide (1 mg,bid)	AZM	10	①②③④⑤⑥
Sun et al. (2024)	62	62	31/31	32/20	6.01 ± 0.65	5.95 ± 0.62	AZM + Budesonide (1 mg,bid)	AZM	7	①②③④⑤⑥
Su, (2020)	50	50	20/30	28/22	6.09 ± 1.22	6.14 ± 1.09	AZM + Budesonide (1 mg,bid)	AZM	7	①②③④⑥
Pan et al. (2023)	60	60	27/23	29/21	5.08 ± 2.25	5.14 ± 2.23	AZM + Budesonide (1 mg,bid)	AZM	7	①②③⑤⑥
Nie, (2022)	43	43	27/16	26/17	3.04 ± 0.18	3.02 ± 0.21	AZM + Budesonide (1 mg,bid)	AZM	14	①③④⑥
Ma and Liu (2024)	82	82	44/38	43/39	5.83 ± 2.11	7.72 ± 2.05	AZM + Budesonide (1 mg,bid)	AZM	14	⑥
Liu and Wang, (2020)	28	28	15/13	17/11	3.6 ± 0.4	3.5 ± 0.6	AZM + Budesonide (1 mg,bid)	AZM	14	①⑥
Liao et al. (2017)	39	39	22/17	23/16	6.54 ± 1.33	6.71 ± 1.38	AZM + Budesonide (bid)	AZM	21	①②③④⑥
Li et al. (2020a)	43	43	26/17	25/18	7.22 ± 2.01	5.21 ± 1.33	AZM + Budesonide (1 mg,bid)	AZM	14	①③⑤
Lei, (2020)	50	50	20/30	28/22	6.09 ± 1.22	6.14 ± 1.09	AZM + Budesonide (1 mg,bid)	AZM	7	①②④⑥
Jin and Tian, (2021)	50	50	28/22	27/23	7.29 ± 0.36	7.43 ± 0.25	AZM + Budesonide (0.5 mg,bid)	AZM	7	⑥
Huang and Song, (2024)	40	40	22/18	21/19	7.02 ± 1.57	6.47 ± 1.56	AZM + Budesonide (0.5 mg,bid)	AZM	7	①③④⑥
Huang et al. (2020)	39	39	25/14	22/17	6.89 ± 1.20	6.92 ± 1.23	AZM + Budesonide (1 mg,bid)	AZM	14	①⑥
Hao and Guo, (2023)	100	100	53/47	52/48	6.9 ± 3.6	6.4 ± 3.2	AZM + Budesonide (1 mg,bid)	AZM	14	①⑤⑥
Fang, (2017)	38	38	22/16	21/17	6.7 ± 1.4	6.5 ± 1.3	AZM + Budesonide (0.5 mg,bid)	AZM	21	①②③④⑥
Cheng, (2021)	40	40	21/19	23/17	6. 45 ± 1.35	6.58 ± 1.46	AZM + Budesonide (1 mg,bid)	AZM	7	①⑥
Chen et al. (2022)	40	40	19/21	21/19	7.01 ± 1.48	6.92 ± 1.54	AZM + Budesonide (0.5 mg,bid)	AZM	14	①②④⑥
Liang and Wang, (2019)	67	67	37/30	38/29	3.1 ± 0.7	3.4 ± 0.7	AZM + Budesonide (0.5 mg,bid)	AZM	7	①②③④⑥
Kang, (2023)	50	50	29/21	25/25	5.69 ± 1.21	5.78 ± 1.65	AZM + Terbutaline (2.5mg–5 mg,bid)	AZM	14	①②③④⑤⑥
Zhao, (2019)	50	50	31/19	30/20	5.39 ± 0.51	5.36 ± 0.5	AZM + Terbutaline (2.5 mg,bid)	AZM	7	①②③④⑥
Xu, (2024)	15	15	7/8	9/6	6.43 ± 2.45	6.52 ± 2.46	AZM + Terbutaline (0.065–0.075 mg/kg,tid)	AZM	7	②③④⑤⑥
Liang, (2024)	25	25	15/10	16/9	3.17 ± 1.82	3.31 ± 1.95	AZM + Terbutaline (2.4 mg,bid)	AZM	14	①②③④⑥
Liang and Huang, (2018)	44	44	25/19	26/18	6.91 ± 2.25	6.73 ± 2.17	AZM + Terbutaline (bid)	AZM	14	④
Liang et al. (2018)	100	100	56/44	58/42	5.36 ± 1.22	5.42 ± 1.37	AZM + Terbutaline (2.5 mg,bid)	AZM	14	②③④⑥
Gong et al. (2017)	51	51	30/21	28/23	7.9 ± 3.5	8.1 ± 3.6	AZM + Terbutaline (2.5mg–5 mg,bid)	AZM	14	①②③④
Chen, (2024)	51	51	25/26	24/27	9.12 ± 1.13	9.11 ± 1.16	AZM + Terbutaline (5 mg,bid)	AZM	7	①⑤⑥
Yu, (2021)	16	16	8/8	9/7	5.68 ± 1.12	5.67 ± 1.11	AZM + Acetylcysteine (0.3g,bid)	AZM	21	①②③④
Zhang, (2023b)	40	40	23/17	22/18	5.1 ± 1.1	4.2 ± 1.2	AZM + Acetylcysteine (0.3g,bid)	AZM + NS	10	①②③
Yao, (2023)	34	34	19/15	18/16	6.00 ± 1.43	6.50 ± 1.41	AZM + Acetylcysteine (0.3g,bid)	AZM	14	①②③④⑥
Jin et al. (2018)	35	35	19/16	18/17	6.2 ± 0.4	6.3 ± 0.3	AZM + Acetylcysteine (0.3g,bid)	AZM + NS	5	①②③④⑥
Cui et al. (2021)	50	50	29/21	27/23	6. 34 ± 5. 48	7. 07 ± 4. 96	AZM + Acetylcysteine (0.3g,bid)	AZM + NS	5	②③④
Cai, (2021)	150	150	82/68	80/70	5.46 ± 1.67	5.56 ± 1.34	AZM + Acetylcysteine (0.3g,bid)	AZM	14	①③④
Bian et al. (2019)	40	40	26/14	24/16	6.08 ± 1.12	6.12 ± 1.11	AZM + Acetylcysteine (0.3g,bid)	AZM	21	①②③④⑥
Xiong and Zhu, (2012)	32	30	16/14	18/14	4 ± 2	4 ± 2	AZM + Budesonide + Salbutamol (1 mL + 0.03 mL,bid)	AZM	14	①
Zhang, (2024)	65	65	34/31	35/30	9.16 ± 1.18	9.22 ± 1.24	AZM + Budesonide + Salbutamol (0.5mg–1 mg + 2.5 mg,bid)	AZM	14	①②③④⑤⑥
Zhang, (2023a)	50	50	27/23	26/24	7.54 ± 1.34	7.52 ± 1.31	AZM + Budesonide + Salbutamol (0.5mg–1 mg + 2.5 mg,bid)	AZM	14	①②③④⑤⑥
Yuan, (2020)	70	70	43/27	40/30	6. 28 ± 3. 36	6. 59 ± 3. 14	AZM + Budesonide + Salbutamol (1 mg + 2.5 mg,bid)	AZM	14	①⑥
Mao, (2017)	33	32	17/16	17/15	—	—	AZM + Budesonide + Salbutamol (1 mg + 2.5 mg,bid)	AZM	7	①⑤
Chen, (2022a)	37	37	20/17	19/18	5.42 ± 2.12	7.51 ± 2.12	AZM + Budesonide + Salbutamol (0.5mg–1 mg + 2.5 mg,bid)	AZM	14	①⑤⑥
Chen, (2023)	42	41	25/17	23/18	7.64 ± 1.55	7.72 ± 1.49	AZM + Budesonide + Salbutamol (1 mg + 2.5 mg,bid)	AZM	14	①②③④⑥
Wang, (2014)	47	47	30/17	28/19	5. 27 ± 1. 34	7. 25 ± 1. 65	AZM + Ambroxol (7.5 mg,bid)	AZM	5–14	②④⑥
Fu et al. (2022)	31	30	19/12	13/17	6.48 ± 1.93	6.51 ± 2.03	AZM + Ambroxol (15 mg,bid)	AZM	7	①
Li, (2018a)	54	54	29/25	30/24	5.9 ± 2.4	4.2 ± 1.7	AZM + Ipratropium + Salbutamol (1.25–2.5 mL,bid)	AZM	14	①③④
Dan, (2018)	41	41	23/18	24/17	5.17 ± 0.68	5.22 ± 0.69	AZM + Ipratropium + Salbutamol (2.5 mL,bid)	AZM	7	①
Ge, (2021)	34	34	19/15	18/14	5.12 ± 1.63	4.52 ± 1.06	AZM + Budesonide + Terbutaline (1 mg + 2.5mg–5 mg,bid)	AZM	7	①②③⑥
Shen et al. (2014)	47	45	27/20	26/19	7. 5 ± 1. 2	7. 6 ± 1. 4	AZM + Budesonide + Terbutaline (1 mg + 2.5 mg,bid)	AZM	7	①③④⑤
Wang, (2024)	40	40	17/23	18/22	6.41 ± 1.01	6.28 ± 0.98	AZM + Salbutamol (2.5 mg,bid)	AZM	7	①②③⑥
Wang, (2018)	29	28	16/13	15/13	8.25 ± 3.42	9.21 ± 2.68	AZM + Terbutaline (2.5 mg,bid)	AZM + Ipratropium + Salbutamol (bid)	7	①②③④⑥
Li et al. (2020b)	51	51	28/23	2,922	7.92 ± 1.57	8.13 ± 2.17	AZM + Terbutaline (2.5mg–5 mg,bid)	AZM	14	①②③④
Li, (2018b)	29	29	18/11	19/10	5.7 ± 4.0	5.4 ± 4.2	AZM + Budesonide + Terbutaline (1 mg + 2.5 mgmg,bid)	AZM	7	①②③④
Yang, (2023)	27	27	16/11	17/10	5.51 ± 1.34	5.48 ± 1.58	AZM + Budesonide + Terbutaline (0.5 mg + 2.5 mg,bid)	AZM	14	①②③④⑤
Huang, (2018)	42	42	23/19	25/17	3.5 ± 0.5	3 ± 0.5	AZM + Budesonide (1 mg,bid)	AZM	7	①②③④
Wang, (2024)	43	43	20/23	21/22	6.37 ± 1.81	6.37 ± 1.81	AZM + Budesonide (0.5 mg,bid)	AZM	7	①③④
Shang, (2018)	42	42	23/19	25/17	6.42 ± 2.37	6.54 ± 2.56	AZM + Terbutaline (2.5mg–5 mg,bid)	AZM	10	①②③④
Li, (2017)	33	32	19/14	18/14	3.5 ± 2.5	4.6 ± 1.5	AZM + Terbutaline (2.5 mg,bid)	AZM	14	①
He, (2017)	50	50	30/20	25/25	4.1 ± 2.0	4.2 ± 2.1	AZM + Terbutaline (2.5mg–5 mg,tid)	AZM	7	①②③④
Liu, (2019)	60	60	32/28	34/26	6.55 ± 1.23	6.78 ± 1.34	AZM + Terbutaline (2.5 mg,bid)	AZM	14	①②③④
Zhang, (2017)	76	72	—	—	—	—	AZM + Budesonide (0.5 mg,qd)	AZM	7	①②③

Note: T, test group; C, control group; M,Male; F, female; NS, Nacl (Placebo); AZM, azithromycin; qd, Once every day; bid, Twice every day; tid, Three times every day ① clinical efficacy rate; ② disappearance time of fever; ③ disappearance time of cough; ④ disappearance time of lung rales; ⑤ pulmonary function; ⑥ total adverse events.

### 3.3 Quality assessment

**FIGURE 2:**
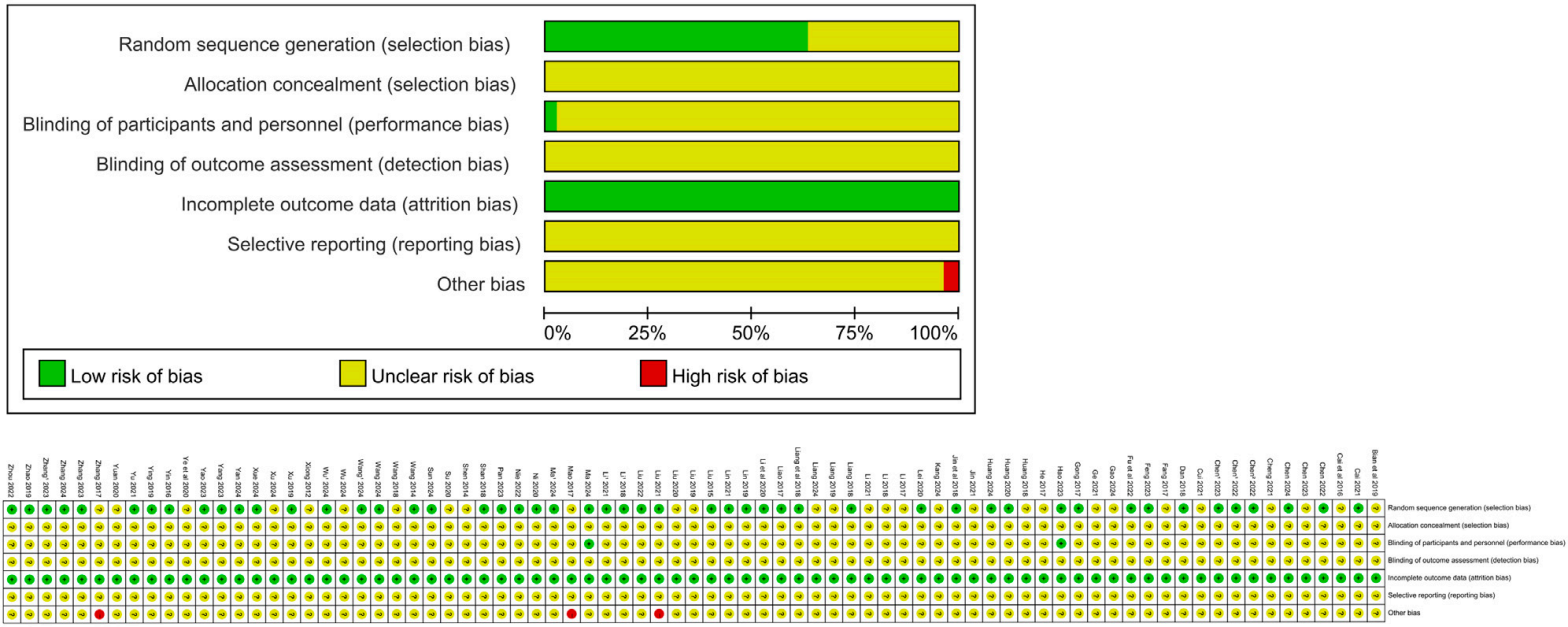
Percentages of items of included articles that produced risks of bias.

According to the evaluation criteria, 40 RCTs utilized random number tables for randomization; 1 RCT employed coin tossing; 1 RCT applied envelope randomization; 2 RCTs used ball touching; 2 RCTs mentioned double-blinding; 4 RCTs adopted lottery methods. These studies were assessed as having a low risk of bias. The remaining 29 RCTs only mentioned randomization, resulting in an assessment of unclear risk. None of the studies mentioned allocation concealment, which was rated as unclear. Blinding was mentioned in only 2 RCTs, which were also assessed as having a low risk of bias; however, these two studies did not provide detailed descriptions of the blinding setting, resulting in the blinding of outcome assessment being rated as unclear. All articles provided complete outcome data. Three RCTs were rated as high risk due to incomplete baseline data. The remaining biases were estimated as unclear due to insufficient details to make a definitive judgment. The risk of bias assessment of the included studies is shown in Figure 2. The quality of evidence for outcome measures was rated as low, with detailed results presented in Additional File 3.

### 3.4 Network geometry

**FIGURE 3:**
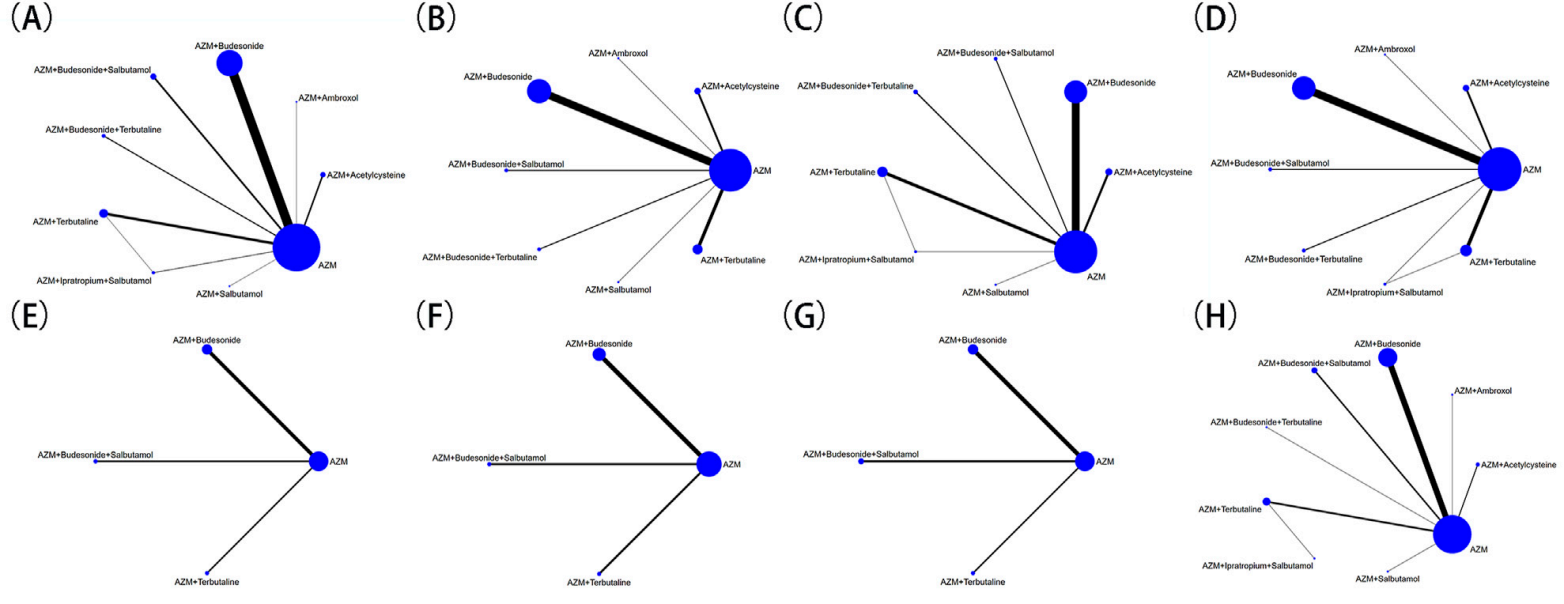
Evidence relationship. **(A)** clinical efficacy rate; **(B)** disappearance time of fever; **(C)** disappearance time of cough; **(D)** disappearance time of pulmonary rales; **(E)** FVC; **(F)** FEV1/FVC; **(G)** PEF; **(H)** adverse events.

The network geometry for outcomes are shown in Figure 3: clinical efficacy rate was reported in 68 studies involving 9 interventions and 6510 patients; the incidence of adverse events were reported in 47 studies involving 9 interventions and 4795 patients; the disappearance time of fever was mentioned in 52 studies involving 8 interventions and 5068 patients; the disappearance time of cough was mentioned in 56 studies involving 8 interventions and 5615 patients; the disappearance time of lung rales was mentioned in 55 studies involving 8 interventions and 5477 patients; and pulmonary function was mentioned in 17 studies involving 4 interventions and 1855 patients. A network diagram of evidence for the presence of a closed loop between interventions requires testing for inconsistency. The results indicated *p* > 0.05, suggesting no evidence of inconsistency in the network model.

### 3.5 Network meta-analysis results

Results from all network meta-analyses are presented in Table 2. In terms of clinical efficacy rate, all nebulized drugs combined with AZM were found to be superior to AZM alone. In terms of the incidence of adverse events, budesonide combined with AZM (OR = 1.68, 95% CI [1.25,2.26]), terbutaline combined with AZM (OR = 2.53, 95% CI [1.18,5.43]) and budesonide + salbutamol combined with AZM (OR = 3.33, 95% CI [1.51,7.34]) were superior to AZM alone. Furthermore, budesonide + terbutaline combined with AZM (OR = 31.29, 95% CI [1.08,902.43]) and terbutaline combined with AZM (OR = 11.2, 95% CI [1.12,112.38]) were superior to ipratropium + salbutamol combined with AZM. Compared to AZM, all treatments except ipratropium + salbutamol combined with AZM and salbutamol combined with AZM demonstrated significant effectiveness in reducing the time to fever, cough, and pulmonary rales. In addition, with the exception of ambroxol combined with AZM, all other treatments were significantly effective than budesonide + salbutamol combined with AZM in reducing fever time; terbutaline combined with AZM (MD = −1.40, 95% CI [-2.76,-0.05]) and ambroxol combined with AZM (MD = −2.78, 95% CI [-5.19,-0.37]) were more effective than ipratropium + salbutamol combined with AZM in the disappearance of pulmonary rales. In terms of lung function, budesonide combined with AZM, terbutaline combined with AZM were significantly superior to AZM. Notably, significant differences were observed only between these specified groups.

**TABLE 2:** Network meta-analysis results of OR and MD with 95%CIs for eight outcomes.

Interventions	Clinical effective rate	Adverse events	Disappearance time of fever	Disappearance time of cough	Disappearance time of lung rales	FVC	FEV1/FVC	PEF
AZM + Budesonide + Terbutaline vs.								
AZM + Ambroxol	0.99 (0.15,6.44)	5.91 (0.39,90.61)	−1.71 (−3.62,0.19)	-	−1.84 (−4.14,0.45)	-	-	-
AZM + Terbutaline	1.09 (0.38,3.13)	2.79 (0.24,32.26)	−0.34 (−1.42,0.73)	−0.78 (−1.75,0.19)	−0.47 (−1.70,0.76)	-	-	-
AZM + Salbutamol	1.11 (0.17,7.08)	10.89 (0.50,236.66)	−0.89 (−2.71,0.94)	−0.39 (−2.18,1.40)	-	-	-	-
AZM + Budesonide	1.17 (0.45,3.06)	4.20 (0.40,43.77)	−0.15 (−1.14,0.84)	−0.32 (−1.20,0.57)	−0.42 (−1.58,0.74)	-	-	-
AZM + Ipratropium + Salbutamol	1.19 (0.33,4.34)	31.29^*^ (1.08,902.43)	-	−0.39 (−1.79,1.02)	−0.93 (−2.67,0.81)	-	-	-
AZM + Budesonide + Salbutamol	1.38 (0.44,4.35)	2.12 (0.18,24.75)	−1.64^*^ (-2.94,-0.33)	−0.41 (−1.62,0.79)	−0.37 (−1.87,1.13)	-	-	-
AZM	6.13^*^ (2.43,15.47)	7.07 (0.69,72.28)	−1.67^*^ (-2.60,-0.73)	−1.96^*^ (-2.80,-1.12)	−1.77^*^ (-2.88,-0.65)	-	-	-
AZM + Terbutaline vs.								
AZM + Salbutamol	1.02 (0.19,5.50)	3.90 (0.45,33.73)	−1.23 (−2.88,0.42)	−0.39 (−2.04,1.27)	-	-	-	-
AZM + Budesonide	1.08 (0.61,1.91)	1.50 (0.66,3.41)	−0.19 (−0.80,0.42)	−0.46 (−1.03,0.10)	−0.05 (−0.68,0.58)	0.14 (−0.53,0.81)	0.05 (−0.25,0.36)	0.17 (−0.43,0.77)
AZM + Ipratropium + Salbutamol	1.10 (0.41,2.91)	11.20^*^ (1.12,112.38)	-	−1.17^*^ (-2.31,-0.03)	−1.40^*^ (-2.76,-0.05)	-	-	-
AZM + Budesonide + Salbutamol	1.27 (0.54,2.97)	1.32 (0.44,3.95)	−1.29^*^ (-2.35,-0.24)	−0.36 (−1.36,0.63)	−0.84 (−1.98,0.30)	0.62 (−0.24,1.48)	0.23 (−0.07,0.53)	0.37 (−0.33,1.06)
AZM	5.64^*^ (3.39,9.38)	2.53^*^ (1.18,5.43)	−2.01^*^ (-2.54,-1.48)	−2.74^*^ (-3.23,-2.24)	−2.24^*^ (-2.77,-1.70)	0.61^*^ (0.04,1.19)	0.45^*^ (0.32,0.59)	0.76^*^ (0.23,1.28)
AZM + Budesonide vs.								
AZM + Ipratropium + Salbutamol	1.02 (0.40,2.60)	7.44 (0.64,85.96)	-	−0.70 (−1.87,0.46)	−1.36 (−2.73,0.02)	-	-	-
AZM + Budesonide + Salbutamol	1.18 (0.57,2.44)	1.98 (0.85,4.59)	−1.49^*^ (−2.45,-0.53)	−0.10 (−1.01,0.81)	−0.79 (−1.85,0.26)	0.47 (−0.25,1.19)	0.17 (−0.21,0.56)	0.20 (−0.33,0.72)
AZM	5.23^*^ (4.03,6.80)	1.68^*^ (1.25,2.26)	−1.82^*^ (-2.12,-1.51)	−2.27^*^ (-2.55,-1.99)	−2.19^*^ (-2.52,-1.85)	0.47^*^ (0.13,0.81)	0.40^*^ (0.12,0.67)	0.59^*^ (0.31,0.86)
AZM + Acetylcysteine vs.								
AZM + Budesonide + Terbutaline	1.27 (0.39,4.07)	6.34 (0.50,80.68)	−0.61 (−1.76,0.53)	−0.49 (−1.51,0.54)	−0.48 (−1.82,0.85)	-	-	-
AZM + Ambroxol	1.25 (0.21,7.41)	1.07 (0.18,6.26)	−2.33^*^ (-4.12,-0.54)		−1.36 (−3.50,0.77)	-	-	-
AZM + Terbutaline	1.38 (0.58,3.30)	2.27 (0.63,8.19)	−0.96^*^ (-1.80,-0.11)	−0.29 (−1.06,0.48)	−0.01 (−0.93,0.90)	-	-	-
AZM + Salbutamol	1.41 (0.24,8.14)	1.72 (0.18,16.56)	−0.27 (−1.97,1.42)	−0.09 (−1.78,1.60)		-	-	-
AZM + Budesonide	1.48 (0.70,3.16)	1.51 (0.52,4.41)	−0.76^*^ (-1.49,-0.03)	−0.17 (−0.82,0.48)	−0.06 (−0.87,0.75)	-	-	-
AZM + Ipratropium + Salbutamol	1.51 (0.48,4.76)	4.93 (0.35,69.01)		−0.87 (−2.15,0.40)	−1.42 (−2.94,0.11)	-	-	-
AZM + Budesonide + Salbutamol	1.75 (0.66,4.67)	2.99 (0.81,10.95)	−2.25^*^ (-3.37,-1.13)	−0.07 (−1.12,0.97)	−0.85 (−2.10,0.39)	-	-	-
AZM	7.77^*^ (3.82,15.80)	1.11 (0.40,3.13)	−1.05^*^ (-1.71,-0.39)	−2.44^*^ (-3.03,-1.85)	−2.25^*^ (-2.99,-1.51)	-	-	-
AZM + Ambroxol vs.								
AZM + Terbutaline	1.10 (0.20,6.09)	2.12 (0.42,10.71)	−1.37 (−3.12,0.37)	-	−1.37 (−3.45,0.70)	-	-	-
AZM + Salbutamol	1.13 (0.11,11.10)	1.84 (0.16,21.86)	−2.60^*^ (-4.88,-0.32)	-	-	-	-	-
AZM + Budesonide	1.19 (0.23,6.20)	1.41 (0.33,6.06)	−1.56 (−3.25,0.13)	-	−1.42 (−3.45,0.61)	-	-	-
AZM + Ipratropium + Salbutamol	1.21 (0.19,7.80)	5.29 (0.32,88.66)	-	-	−2.78^*^ (-5.19,-0.37)	-	-	-
AZM + Budesonide + Salbutamol	1.40 (0.24,8.19)	2.78 (0.54,14.28)	0.08 (−1.82,1.97)	-	−2.22 (−4.46,0.02)	-	-	-
AZM	6.21^*^ (1.22,31.78)	1.20 (0.29,5.00)	−3.38^*^ (-5.04,-1.72)	-	−3.61^*^ (-5.61,-1.61)	-	-	-
AZM + Salbutamol vs.								
AZM + Budesonide	1.05 (0.21,5.35)	2.59 (0.34,19.92)	−1.04 (−2.63,0.56)	−0.08 (−1.68,1.53)	-	-	-	-
AZM + Ipratropium + Salbutamol	1.07 (0.17,6.76)	2.87 (0.12,67.56)	-	−0.78 (−2.73,1.16)	-	-	-	-
AZM + Budesonide + Salbutamol	1.24 (0.22,7.09)	5.13 (0.59,44.82)	−2.52^*^ (-4.33,-0.71)	−0.02 (−1.83,1.78)	-	-	-	-
AZM	5.52^*^ (1.11,27.43)	1.54 (0.20,11.59)	−0.78 (−2.34,0.78)	−2.35^*^ (-3.93,-0.77)	-	-	-	-
AZM + Ipratropium + Salbutamol vs.								
AZM + Budesonide + Salbutamol	1.16 (0.37,3.57)	14.73^*^ (1.14,189.50)	-	−0.80 (−2.22,0.62)	−0.56 (−2.23,1.11)	-	-	-
AZM	5.13^*^ (2.09,12.63)	4.42 (0.39,50.20)	-	−1.57^*^ (-2.70,-0.44)	−0.83 (−2.17,0.51)	-	-	-
AZM + Budesonide + Salbutamol vs.								
AZM	4.44^*^ (2.25,8.76)	3.33^*^ (1.51,7.34)	−3.30^*^ (-4.21,-2.39)	−2.37^*^ (-3.24,-1.51)	−1.39^*^ (-2.40,-0.39)	−0.00 (−0.64,0.63)	0.22 (−0.04,0.49)	0.39 (−0.06,0.84)

Note: * Compared between the two groups, *p* < 0.05; FVC, forced vital capacity; FEV1/FVC, forced expiratory volume in one second/forced vital capacity; PEF, peak expiratory flow.

### 3.6 Ranking results based on SUCRA

The SUCRA values for the results are presented in Table 3. In terms of clinical efficacy rate, the probability of acetylcysteine combined with AZM being the most effective intervention for clinical efficacy was 76.7%. This was followed by budesonide + terbutaline combined with AZM (SUCRA: 61.4%) and ambroxol combined with AZM (SUCRA: 59.9%). In terms of the incidence of adverse events, budesonide + terbutaline combined with AZM (SUCRA: 88.8%) was the most effective intervention, followed by budesonide + salbutamol combined with AZM (SUCRA: 83.1%) and terbutaline combined with AZM (SUCRA: 74%). For the disappearance time of fever, the top three treatments were budesonide + salbutamol combined with AZM (SUCRA: 92.2%), ambroxol combined with AZM (SUCRA: 91.2%), and terbutaline combined with AZM (SUCRA: 63.4%). In terms of the disappearance time of cough, terbutaline combined with AZM (SUCRA 86.8%) ranked highest, followed by acetylcysteine combined with AZM (SUCRA: 67.8%) and budesonide + salbutamol combined with AZM (SUCRA: 62.6%). For the disappearance time of pulmonary rales, ambroxol combined with AZM (SUCRA: 94.6%) was the most effective treatment, followed by terbutaline combined with AZM (SUCRA: 69.3%) and acetylcysteine combined with AZM (SUCRA: 68.9%). Lastly, terbutaline combined with AZM (SUCRA: 85.1%, 72.4%, 85.4%, respectively) and budesonide combined with AZM (SUCRA: 74.5%, 85.7%, 68.2%, respectively) ranked highest for improvements in pulmonary function parameters, including FVC, FEV1/FVC, and PEF.

**TABLE 3:** SUCRA results of eight outcomes.

Interventions	Clinical effective rate	Adverse events	Disappearance time of cough fever	Disappearance time of cough	Disappearance time of lung rales	FVC	FEV1/FVC	PEF
AZM + Budesonide + Terbutaline	61.4	88.8	48.2	39.2	47	-	-	-
AZM + Terbutaline	56.3	74	63.4	86.8	69.3	85.1	72.4	85.4
AZM + Budesonide	49.9	56.5	54.3	55.1	65.8	74.5	85.7	68.2
AZM + Ambroxol	59.9	43	91.2	-	94.6	-	-	-
AZM + Salbutamol	55.5	26.9	22.6	61.7	-	-	-	-
AZM + Acetylcysteine	76.7	38.2	25.9	67.8	68.9	-	-	-
AZM + Ipratropium + Salbutamol	49.5	9.3	-	26.8	19.5	-	-	-
AZM + Budesonide + Salbutamol	40.3	83.1	92.2	62.6	33.1	23.2	40.1	44.8
AZM	0.4	30.1	2.2	0.1	1.8	17.2	1.8	1.6

Note: AZM, azithromycin; FVC, forced vital capacity; FEV1/FVC, forced expiratory volume in one second/forced vital capacity; PEF, peak expiratory flow.

### 3.7 Cluster analysis

**FIGURE 4:**
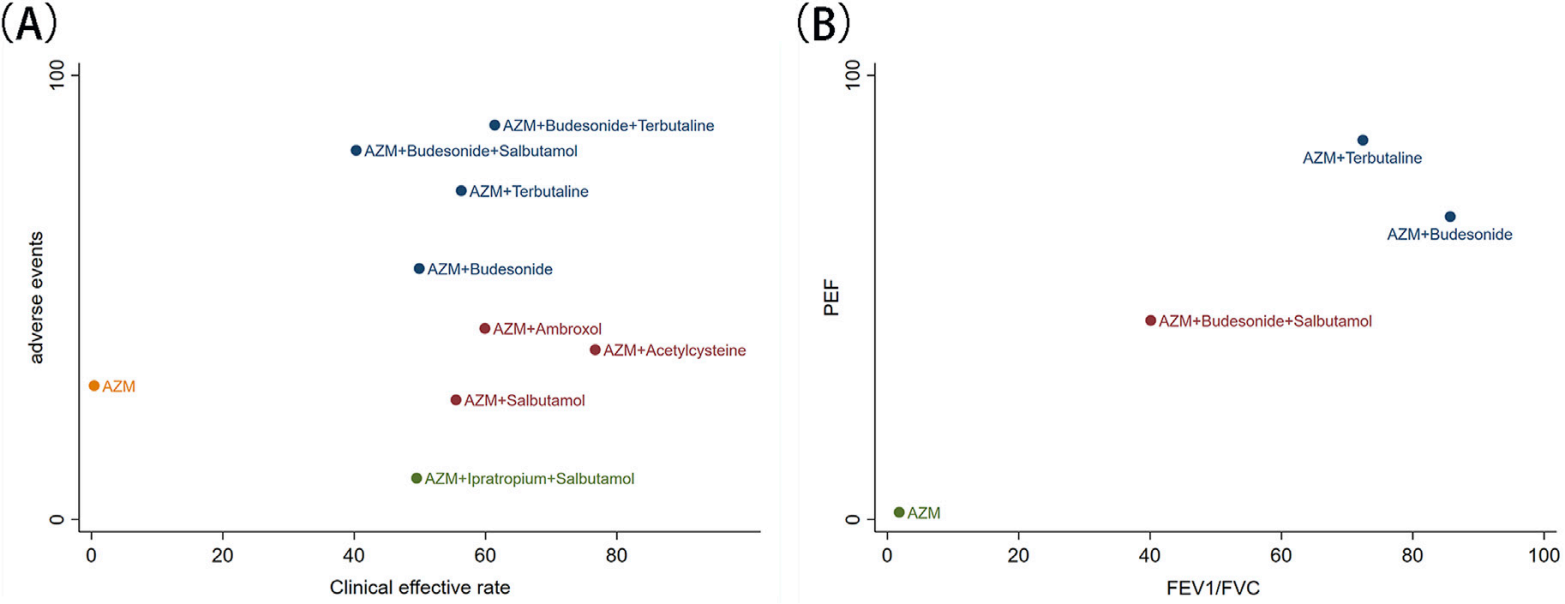
Cluster analysis. **(A)** clinical efficacy rate and adverse events; **(B)** FEV1/FVC and PEF.

In this study, two-group cluster analysis was used, including clinical efficacy rate and the incidence of adverse events, as well as FEV1/FVC and PEF. The results are shown in Figure 4. Through cluster comprehensive analysis, the combination of budesonide + terbutaline with AZM demonstrated favorable clinical efficacy and safety. Furthermore, the combination of budesonide with AZM and terbutaline with AZM improved pulmonary function.

### 3.8 Publication bias

**FIGURE 5:**
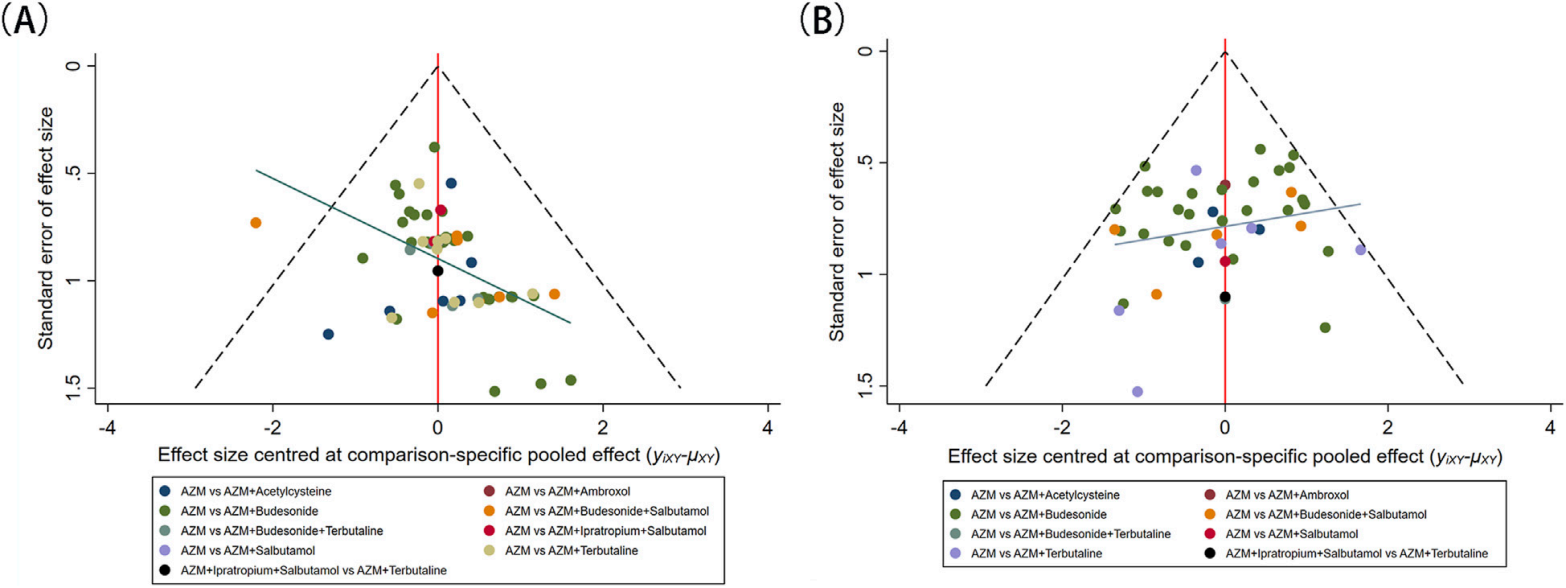
Funnel plots. **(A)** clinical efficacy rate; **(B)** adverse events.

The comparative-corrected funnel plot results indicated that the funnel plot for the clinical efficacy rate exhibited asymmetry, implying potential small sample effect. In contrast, the funnel plot for the incidence of adverse events appeared largely symmetrical on both the left and right sides, suggesting a minimal risk of publication bias. The results are shown in Figure 5.

### 3.9 Subgroup and sensitivity analysis

Analyses revealed heterogeneity in the disappearance time of fever, cough, lung rales, and pulmonary function. However, results from subgroup analyses based on age and treatment duration indicated that this heterogeneity remained unchanged (Supplementary Figures S1, S2). Sensitivity analyses were performed on the clinical efficacy rate, the incidence of adverse events, the disappearance time of fever, cough, and lung rales, as well as pulmonary function (FEV1/FVC). The results showed that the sensitivity analyses for all outcomes were stable, with no significant bias detected, thereby affirming the stability of the meta-analysis results (Supplementary Figure S3).

## 4 Discussion

Nebulized inhalation therapy has demonstrated distinct advantages in treating MPP. The appropriate selection of nebulized medications can accelerate the resolution of clinical symptoms and reduce the risk of severe infections. This study represents the first application of network meta-analysis in evaluating the differences in efficacy and safety among various nebulized drugs combined with AZM for treating non-severe MPP in children. The results indicate that, compared to AZM alone, all nebulized drugs combined with AZM improved the overall clinical efficacy in children with non-severe MPP to varying extents, shortened the duration of symptoms, enhanced pulmonary function, and did not increase the incidence of adverse events. SUCRA ranking and cluster analysis results revealed that the combination of budesonide + terbutaline with AZM emerged as the optimal intervention in terms of both efficacy and safety. Additionally, ambroxol combined with AZM was particularly effective in alleviating clinical symptoms such as fever and lung rales, while either terbutaline or budesonide combined with AZM significantly improved lung function.

Among the nebulized drugs included in this study, budesonide is a glucocorticoid with anti-inflammatory properties that reduces airway spasm and exerts immunomodulatory effects (Li et al., 2017). The results demonstrated that, compared to AZM, the combination of budesonide and AZM significantly reduced the incidence of adverse events. The SUCRA ranking indicates that nebulized treatments containing budesonide (budesonide + terbutaline, budesonide + salbutamol) were more effective in reducing adverse reactions compared to other drugs. On one hand, budesonide may alleviate airway inflammation and mucus secretion through its anti-inflammatory effects, thus reducing adverse reactions associated with inflammatory responses (Liu et al., 2022). On the other hand, it might indirectly lower the overall risk of adverse reactions by shortening the disease course or reducing other complications. This suggests that, in the short-term treatment of non-severe MPP, the benefits of nebulized budesonide may exceed the potential risks. However, in clinical practice, it is still necessary to determine whether to use budesonide and other glucocorticoid drugs in combination based on the specific conditions of each patient. Notably, both terbutaline and salbutamol are short-acting β2 agonists that improve ventilation and reduce airway inflammation by relaxing bronchial smooth muscle and inhibiting the release of inflammatory mediators from mast cells (Licari et al., 2024). However, study (Motazedian et al., 2010) has shown that terbutaline exhibits higher selectivity, fewer side effects, and longer duration of action compared to salbutamol. Our findings support this view, indicating that nebulized terbutaline generally provides superior therapeutic efficacy to salbutamol.

Additionally, both ambroxol and acetylcysteine are classified as expectorants that promote sputum expectoration by reducing mucus viscosity and enhancing ciliary motility (Ahmadi et al., 2024; Chen et al., 2023; Deretic and Timmins, 2019). This mechanism accelerates the alleviation of clinical symptoms and improves therapeutic efficacy; however, in terms of safety, both drugs are ranked relatively low. Overall, the combination of budesonide + terbutaline with AZM demonstrates superior efficacy and safety compared to other nebulized drugs. This combination establishes a triple synergistic mechanism of “anti-infection, anti-inflammation, and improved ventilation,” effectively enhancing the cure rate, reducing the risk of severe illness, and minimizing the occurrence of adverse events. Furthermore, AZM possesses immunomodulatory properties that can inhibit neutrophil chemotaxis and cytokine release, which synergize with the anti-inflammatory effects of nebulized corticosteroids such as budesonide to significantly ameliorate airway inflammation. This may clarify the observed differences in efficacy and safety between budesonide-based nebulized therapy and other nebulized treatment modalities in this study. These findings provide an evidence-based rationale and theoretical foundation for selecting appropriate nebulized medications in the clinical treatment of non-severe MPP in children.

In comparison to the relevant meta-analyses (Zhao et al., 2024; Sheng et al., 2024), several important points warrant attention. First, these studies exhibited inconsistencies in their inclusion criteria for MPP patients, failing to differentiate between severe and non-severe cases, which may introduce confounding factors and bias in efficacy assessments. Second, the criteria for incorporating interventions in these studies lacked rigor; for instance, the use of antitussives, expectorants, and other oral medications could confound clinical outcomes. In contrast, our study adopted a well-defined PICOS framework to rigorously delineate both the study population and interventions, excluding severe and refractory infections while minimizing potential confounding effects from adjunct medications. This approach mitigates clinical and methodological heterogeneity. Furthermore, unlike studies limited to pairwise comparisons, our research provides a comprehensive evaluation of all nebulized drug treatment regimens, enabling clinicians to identify the relatively optimal therapeutic strategy among numerous RCTs.

This study has several limitations, which are primarily reflected in the following aspects: 1) The quality of the included literature was generally low. Some original articles in our selected studies lacked detailed descriptions of the random sequence generation method (e.g., computerized randomization, coin toss) and allocation concealment mechanism (e.g., sealed envelope, central randomization system), and did not explicitly report the implementation of blinding. This absence of detail may increase the risk of bias. 2) The range of included drugs was not comprehensive; with the exception of one study, all others represent indirect comparisons. Indirect evidence may contribute to statistical heterogeneity due to variations in trial populations or administered doses, resulting in diminished confidence in the accuracy of the results. 3) The number of studies on specific drugs remains limited, including albuterol (1 study), ambroxol (2 studies), and albuterol combined with ipratropium bromide (2 studies). Moreover, treatment durations of the included studies vary significantly, ranging from 5 to 21 days 4) All included RCTs were conducted in China, which limits the applicability of the results to other settings with different healthcare systems or clinical practices. 5) The absence of a gold standard could influence endpoint outcomes; although established criteria exist for pulmonary function assessment, the limited number of studies reporting this metric necessitates cautious interpretation of the findings.

Adjuvant therapy with nebulized drugs can enhance the efficacy of treatment for non-severe MPP in children, shorten the duration of symptoms, improve pulmonary function, and without increasing the incidence of adverse events. Although the combined use of nebulized drugs may raise the per-treatment cost, their effectiveness in shortening the course of the disease and improving prognosis may offset the initial expenses. A study demonstrated that combining aerosol inhalation in the treatment of bronchopneumonia significantly reduced both direct medical costs and indirect costs such as transportation and nursing expenses (Zhu et al., 2023). Furthermore, an efficient nebulized treatment regimen reduces the risk of drug resistance and avoids additional costs associated with subsequent treatment escalation. Among the nine interventions studied, the combination of budesonide + terbutaline with AZM demonstrated favorable safety and efficacy profiles, providing a valuable reference for clinical medication. However, due to the limitations in this study, future higher-quality research across different regions is necessary to support global recommendations.

## Data Availability

The original contributions presented in the study are included in the article/Supplementary Material, further inquiries can be directed to the corresponding authors.
