# Supplementary material for: Efficacy and safety of nebulized drugs in the treatment of non-severe mycoplasma pneumoniae pneumonia in children - a network meta-analysis
Source: Front Pharmacol. 2025 Sep 2;16:1587152. doi: 10.3389/fphar.2025.1587152 (PMC12436391; doi:10.3389/fphar.2025.1587152)
Supplement: Supplementary file 1 [file DataSheet2.PDF]

Supplementary Figure S2

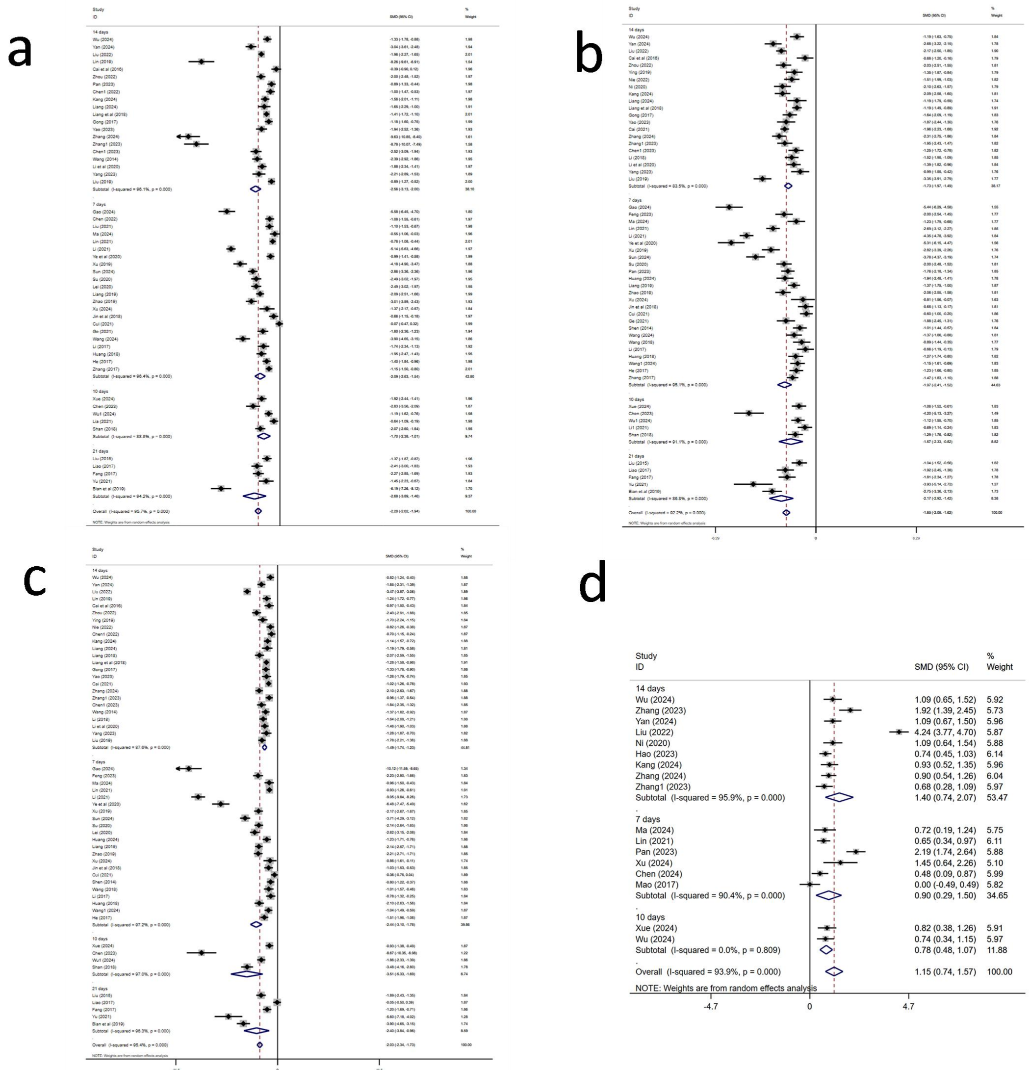

Figure S2: Subgroup analyses of the treatment course(7 days,10days, 14days or 21 days). (a) disappearance time of fever, (b) disappearance time of cough, (c) disappearance time of lung rales, and (d) pulmonary function (FEV1/FVC).
