# Supplementary material for: Efficacy and safety of nebulized drugs in the treatment of non-severe mycoplasma pneumoniae pneumonia in children - a network meta-analysis
Source: Front Pharmacol. 2025 Sep 2;16:1587152. doi: 10.3389/fphar.2025.1587152 (PMC12436391; doi:10.3389/fphar.2025.1587152)
Supplement: Supplementary file 2 [file DataSheet4.PDF]

Author(s):
Question: AZM combined with nebulized drug inhalation therapy compared to AZM sequential therapy alone or combined nebulized drugs for Non-Severe Mycoplasma Pneumoniae Pneumonia in Children
Setting:
Bibliography:

Certainty assessment							№ of patients		Effect		Certainty	Importance
№ of studies	Study design	Risk of bias	Inconsistency	Indirectness	Imprecision	Other considerations	AZM combined with nebulized drug inhalation therapy	AZM sequential therapy alone or combined nebulized drugs	Relative (95% CI)	Absolute (95% CI)		
**clinical efficacy rate**												
68	randomised trials	serious[a]	not serious	not serious	not serious	publication bias strongly suspected[b,c]	3128/3263 (95.9%)	2607/3247 (80.3%)	**OR 5.70** (4.70 to 6.92)	**156 more per 1,000** (from 147 more to 163 more)	⨁⨁◯◯ Low[a,b,c]	CRITICAL
**disappearance time of fever**												
52	randomised trials	serious[a]	serious[d]	not serious	not serious	publication bias strongly suspected[b,c]	2537	2531	-	SMD **2.28 SD lower** (2.62 lower to 1.94 lower)	⨁◯◯◯ Very low[a,b,c,d]	IMPORTANT
**disappearance time of cough**												
56	randomised trials	serious[a]	serious[d]	not serious	not serious	publication bias strongly suspected[b,c]	2816	2799	-	SMD **1.85 SD lower** (2.08 lower to 1.62 lower)	⨁◯◯◯ Very low[a,b,c,d]	IMPORTANT
**disappearance time of pulmonary rales**												
56	randomised trials	serious[a]	serious[d]	not serious	not serious	publication bias strongly suspected[b,c]	2745	2732	-	SMD **2.03 SD lower** (2.34 lower to 1.73 lower)	⨁◯◯◯ Very low[a,b,c,d]	IMPORTANT
**FEV1/FVC**												
17	randomised trials	serious[a]	serious[d]	not serious	not serious	publication bias strongly suspected[b,c]	928	927	-	SMD **1.22 SD higher** (0.8 higher to 1.65 higher)	⨁◯◯◯ Very low[a,b,c,d]	IMPORTANT
**adverse events**												
47	randomised trials	serious[a]	not serious	not serious	not serious	publication bias strongly suspected[c]	181/2402 (7.5%)	323/2393 (13.5%)	**OR 0.52** (0.43 to 0.62)	**60 fewer per 1,000** (from 72 fewer to 47 fewer)	⨁⨁◯◯ Low[a,c]	CRITICAL

CI: confidence interval; **OR:** odds ratio; **SMD:** standardised mean difference

Explanations

a. Most studies lacke clear allocation concealment and blinding during implementation
b. the funnel plot for the outcome measure exhibited asymmetry
c. Some studies have smal-sample sizes
d. Large heterogeneity across studies
