# Supplementary material for: Efficacy and safety of nebulized drugs in the treatment of non-severe mycoplasma pneumoniae pneumonia in children - a network meta-analysis
Source: Front Pharmacol. 2025 Sep 2;16:1587152. doi: 10.3389/fphar.2025.1587152 (PMC12436391; doi:10.3389/fphar.2025.1587152)
Supplement: Supplementary file 3 [file DataSheet6.docx]

**The definition of clinical response**

**Failure** is defined as no improvement or worsening of symptoms after treatment;

**Improvement** is characterized by a significant alleviation of cough and other symptoms, along with positive changes observed in chest X-ray results;

**Cure** is indicated by the complete resolution of all symptoms following treatment, with chest X-ray results demonstrating normalization.

**Overall response rate** = (number of cured patients + number of improved patients)/total number of patients×100%.
