# Supplementary material for: Efficacy and safety of nebulized drugs in the treatment of non-severe mycoplasma pneumoniae pneumonia in children - a network meta-analysis
Source: Front Pharmacol. 2025 Sep 2;16:1587152. doi: 10.3389/fphar.2025.1587152 (PMC12436391; doi:10.3389/fphar.2025.1587152)
Supplement: Supplementary file 4 [file DataSheet5.docx]

**Pubmed：**

#1 ((((((((((((Mycoplasma Pneumonia[Title/Abstract]) OR (Mycoplasma Pneumonias[Title/Abstract])) OR (Pneumonias, Mycoplasma[Title/Abstract])) OR (Pneumonia, Primary Atypical[Title/Abstract])) OR (Atypical Pneumonia, Primary[Title/Abstract])) OR (Atypical Pneumonias, Primary[Title/Abstract])) OR (Pneumonias, Primary Atypical[Title/Abstract])) OR (Primary Atypical Pneumonia[Title/Abstract])) OR (Primary Atypical Pneumonias[Title/Abstract])) OR (Mycoplasma pneumoniae Infection[Title/Abstract])) OR (Mycoplasma pneumoniae Infections[Title/Abstract])) OR (Mycoplasma ovipneumoniae Infection[Title/Abstract])) OR (Mycoplasma ovipneumoniae Infections[Title/Abstract])) OR (Mycoplasma dispar Infection[Title/Abstract])) OR (Mycoplasma dispar Infections[Title/Abstract])) OR ("Pneumonia, Mycoplasma"[Mesh])) OR (mycoplasma pneumoniae pneumonia[Title/Abstract])

#2 (((atomization[Title/Abstract]) OR (Nebulization therapy[Title/Abstract])) OR (aerosol inhalation[Title/Abstract])) OR (nebulized[Title/Abstract])

#3 ("Child"[Mesh]) OR (Children[Title/Abstract])

#4 #1 AND #2 AND #3

**Embase:**

#12. #9 AND #10 AND #11

#11. #7 OR #8

#10. #3 OR #4 OR #5 OR #6

#9. #1 OR #2

#8. children:ab,ti

#7. 'child'/exp OR 'child'

#6. nebulized:ab,ti

#5. aerosol AND inhalation:ab,ti

#4. nebulization AND therapy:ab,ti

#3. 'nebulization'/exp OR 'nebulization'

#2. mycoplasma AND pneumoniae AND pneumonia:ab,ti

#1. 'mycoplasma pneumoniae'/exp

**Web of science:**

#1 (TS=(Mycoplasma Pneumonia )) OR TS=(mycoplasma pneumoniae pneumonia)

#2 (((TS=(atomization)) OR TS=(Nebulization therapy)) OR TS=(aerosol inhalation)) OR TS=(nebulized)

#3 (TS=(Child)) OR TS=(Children)

#3 AND #2 AND #1

**Cochrane:**

#1 (mycoplasma pneumoniae pneumonia):ti,ab,kw OR (Mycoplasma Pneumonia):ti,ab,kw OR (Pneumonia, Mycoplasma):ti,ab,kw

#2 (Child):ti,ab,kw OR (Children):ti,ab,kw

#3 #1 AND #2

**CNKI:**

SU = ('children' + 'child' ) AND SU = ('Mycoplasma pneumoniae' + 'Mycoplasma pneumonia') AND SU = ('aerosol' + 'aerosol inhalation' + 'aerosol therapy')

**VIP:**

(M=children OR M=child) AND (M=Mycoplasma pneumoniae OR M=Mycoplasma pneumonia) AND (M=aerosol OR M=aerosol inhalation OR M=aerosol therapy)

**Wang Fang:**

Title or Keywords: (children OR child) AND Title or Keywords: (Mycoplasma pneumoniae OR Mycoplasma pneumonia) AND Title or Keywords: (aerosol OR aerosol inhalation OR aerosol therapy)

**CBM:**

("children"[Title: intelligent] OR "child"[Title: intelligent] ) AND ("Mycoplasma pneumoniae"[Title: intelligent] OR "Mycoplasma pneumonia"[Title: intelligent]) AND ("aerosol"[Title: intelligent] OR "aerosol inhalation"[Title: intelligent])
