# Supplementary material for: Efficacy and safety of nebulized drugs in the treatment of non-severe mycoplasma pneumoniae pneumonia in children - a network meta-analysis
Source: Front Pharmacol. 2025 Sep 2;16:1587152. doi: 10.3389/fphar.2025.1587152 (PMC12436391; doi:10.3389/fphar.2025.1587152)
Supplement: Supplementary file 5 [file DataSheet3.PDF]

Supplementary Figure S3

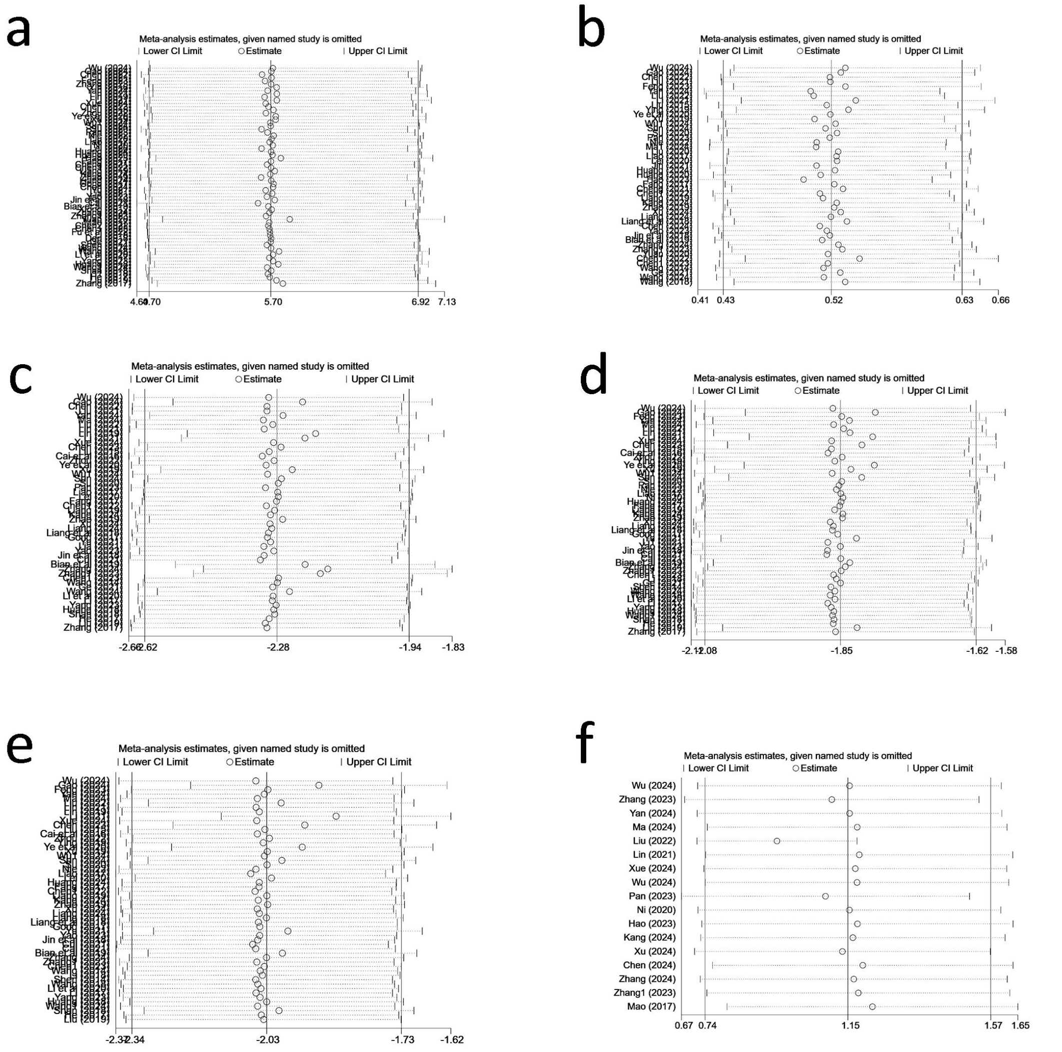

Figure S3: Sensitivity analyses were performed on (a) clinical efficacy rate, (b) total adverse events, (c) disappearance time of fever, (d) disappearance time of cough, (e) disappearance time of lung rales, and (f) pulmonary function (FEV1/FVC).
