# Supplementary material for: Efficacy and safety of nebulized drugs in the treatment of non-severe mycoplasma pneumoniae pneumonia in children - a network meta-analysis
Source: Front Pharmacol. 2025 Sep 2;16:1587152. doi: 10.3389/fphar.2025.1587152 (PMC12436391; doi:10.3389/fphar.2025.1587152)
Supplement: Supplementary file 6 [file DataSheet1.PDF]

Supplementary Figure S1

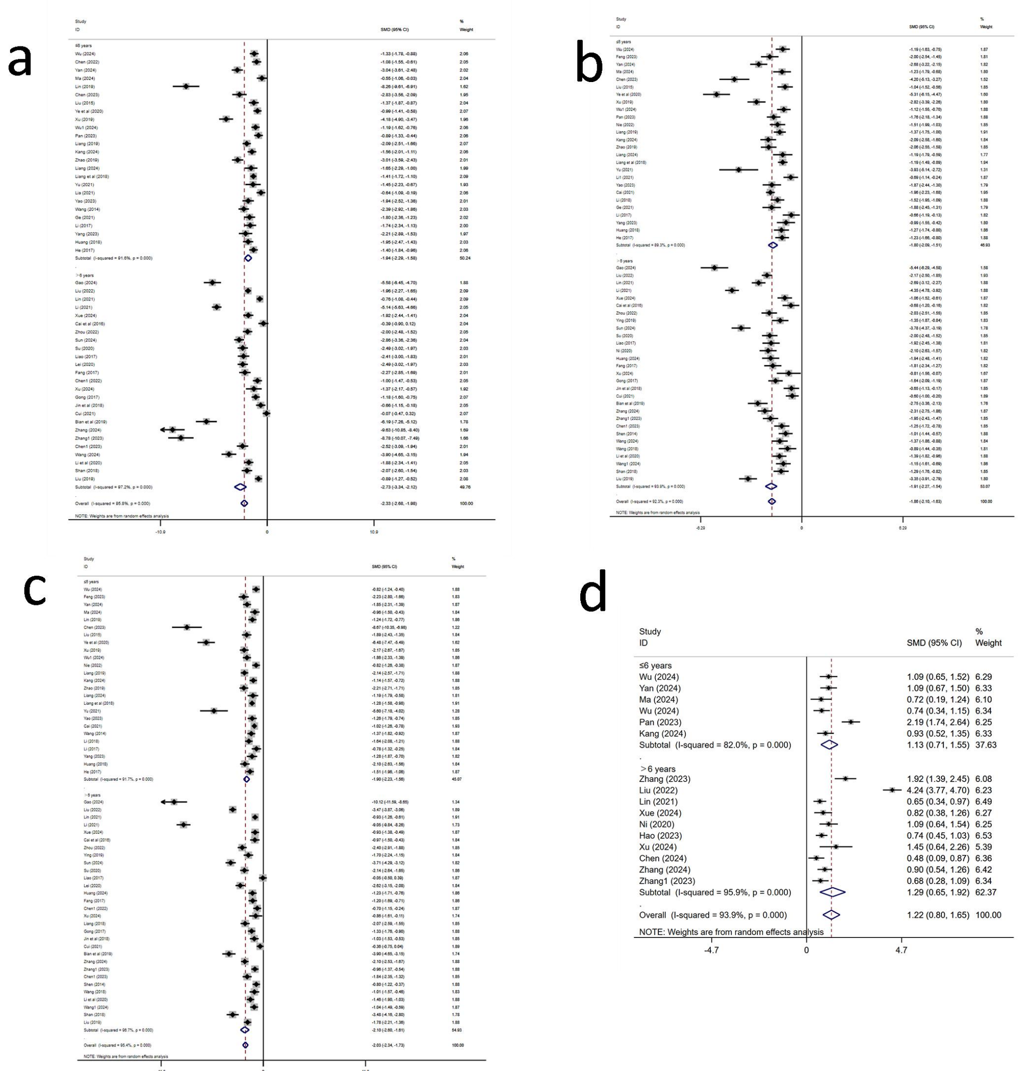

Figure S2: Subgroup analyses of the age(≤6 years or ＞6 years). (a) disappearance time of fever, (b) disappearance time of cough, (c) disappearance time of lung rales, and (d) pulmonary function (FEV1/FVC).
